# Precise determination of Young’s modulus of amorphous CuZr/nanocrystalline Cu multilayer via nanoindentation

**DOI:** 10.1557/s43578-023-01057-y

**Published:** 2023-06-18

**Authors:** Alice Lassnig, Stanislav Zak

**Affiliations:** Erich Schmid Institute of Materials Science, Austrian Academy of Sciences, 8700 Leoben, Austria

**Keywords:** Amorphous CuZr, Elastic properties, Nanocrystalline Cu, Nano-indentation, Substrate effect, Thin film

## Abstract

Extracting mechanical data of thin films on rigid substrates using nanoindentation is compromised by the mechanical properties of underlying substrates, which may falsify the obtained results. With ongoing miniaturization, the substrate influence becomes more pronounced. In this study we present an experimental approach to extract the true Young’s modulus of crystalline-amorphous multilayers by means of nanoindentation. We used 1 µm thick multilayers comprised of amorphous CuZr and nanocrystalline Cu. All films were deposited onto two rigid substrate types with Young’s moduli below and above the ones expected for the deposits (film-to-substrate hardness and elastic moduli ratios between 0.3 to 1.1 and 0.6 to 1.5, respectively). Linear extrapolation of indentation data to zero indentation depth allows to precisely determine the real film’s Young’s modulus. Same investigations were performed on monolithic Cu and CuZr films of same thickness. While the hardness values change with the variation of the bilayer thickness of the multilayer structures, the Young’s modulus is not affected by the interfaces.

**Graphical abstract:**

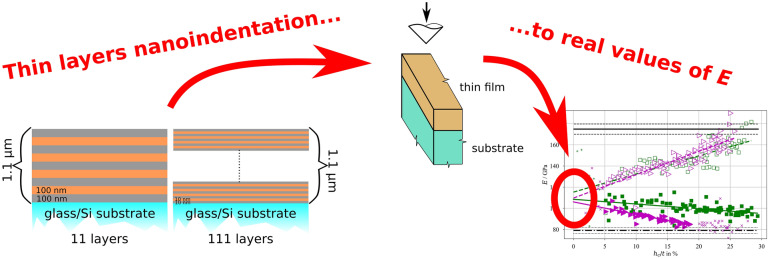

## Introduction

Nanoindentation has become the experimental method of choice to rapidly assess the mechanical properties of thin films and multilayered structures attached to rigid substrates. This technique is rather easy to use requiring no elaborate sample preparation and multiple data points can be gathered in reasonable time delivering reliable results with sound statistics. However, the emergence of small-scaled structures and ongoing miniaturization of thin film and multilayered structures in cutting edge technologies (such as microelectronic devices) have pushed nanoindentation to its limits. With decreasing film thickness of the probed structures, the obtained results are increasingly compromised by the underlying substrates [1–5]. While hardness measurements remain less sensitive to the substrate effect, the measurements of elastic moduli are strongly affected. The elastic field beneath the indenter is not confined to the film thickness itself but it is a long-range field extending into the substrate. This is more pronounced at low film thicknesses, especially when a large elastic mismatch between probed layer and substrate is present.

Doerner et al. [5] developed a model that attempted to account for the influence of the substrate compliance by including a term for the substrate in the reduced modulus equation, but their model is only valid for the specific thin film-substrate materials combination presented in their study. King [6] adapted and modified this model [5], however, it includes a numerically determined scaling parameter, which cannot be obtained in a fast and straightforward way and the model was not generalized to other material systems. Moreover, several different case-specific strategies were adapted for nanoindentation measurements to account for the substrate effect, such as [7], but their generalization might be questionable. More recently, the possibility of larger than previously expected substrate influence has also been confirmed and formulated by Bull et al. [3] and further developed by Zak et al. [4], where an experimental routine of indents into Mo and MoTa thin films (*t* = 1; 2 μm) on Si substrates in combination with finite element simulations were used as a guideline to improve the reliability of nanoindentation results of thin films attached to rigid substrates. This proved that the elastic modulus has to be determined as a function of contact depth for a wide range of indentation depths.

In pursuit of optimized mechanical and functional properties of amorphous materials, nanolaminates composed of alternating amorphous and nanocrystalline sublayers [8] have been proven to be a promising strategy to counteract the main disadvantage of amorphous metals—namely strain softening and catastrophic failure due to shear banding as the main deformation mechanism due to the lack of long-range order [9]. It is well-known that the introduction of a soft, ductile crystalline phase enhances plastic deformation and prevents abrupt failure. Furthermore, it has been shown that in amorphous-nanocrystalline multilayers, nanocrystalline layers can arrest shear bands initiating in amorphous layers [10, 11], and therefore such nanolaminates allow to combine the high strength and ductility of both material classes. In the field of amorphous-nanocrystalline multilayer materials, amorphous CuZr and nanocrystalline Cu nanolaminates have been extensively studied using (ex situ) indentation-based techniques and in situ pillar compression testing [12] to reveal how interface density and individual layer thickness of the nanocrystalline [10] and amorphous sublayers [13] influence the deformation mechanisms. While the above-mentioned studies focused on hardness, plastic deformation, and microstructure of crystalline-amorphous nanolaminates, a proper measurement of thin nanolaminate multilayers’ Young’s modulus was tackled only sporadically. In contrast to that Young’s moduli of single CuZr amorphous thin films compared to their bulk counterparts have been determined experimentally using AFM cantilevers and free-standing films as a function of composition in [14], and their values vary between 40 and 90 GPa (depending on the Cu at. %). Additionally, the Young’s modulus of CuZr as a thin film metallic glass as compared to melt-spun ribbons were probed using nanoindentation in [15], leading to values of 114.3 ± 2.2 GPa and 106.7 ± 6.9 GPa for thin film and ribbon, respectively. In the same study the hardness was reported as 3.9 ± 0.163 GPa and 7.5 ± 0.884 GPa for thin film and ribbon, respectively, for the Cu_50_Zr_50_ composition.

In the present work we studied the mechanical behavior of crystalline-amorphous multilayers as a function of their bilayer thickness (Λ = 20 nm vs. 200 nm) using nanoindentation. A specific focus is set on determining the true Young’s moduli as a function of bilayer thickness. The experiments are designed such that the amount of nanocrystalline and amorphous phase are kept identical among the multilayer architectures. Therefore, we can directly unravel how the interface density in the crystalline-amorphous nanolaminates and the underlying substrates influence the mechanical properties. To determine the substrate effect more accurately, two systems with different rigid substrate types are compared—one expected to be more compliant (glass) and one stiffer (Si) than the multilayer structure.

With known elastic modulus of the substrate, the substrate-influenced measurements can be rooted-out by an extrapolation toward *h*_c_ = 0, similarly to measurements presented by Lorenz et al. [16]. However, there are numerous ways how to approach such data extrapolation and a lot of them might be case-specific. On the other hand, combining the extrapolation procedure with two different material systems (same film/multilayer with different substrates), whereas the multiple substrates approach is well in line with results from [17] where a deep analysis was performed on nanoindentation of Al and W thin films on glass and Si substrates, leading to comparison of hard-soft and soft-hard bi-material systems, can lead to more exact and fast solution of an extrapolation, when the real value at *h*_c_ = 0 is approached from two different sides.

## Results

### Multilayer characterization (TEM)

**Figure 1:**
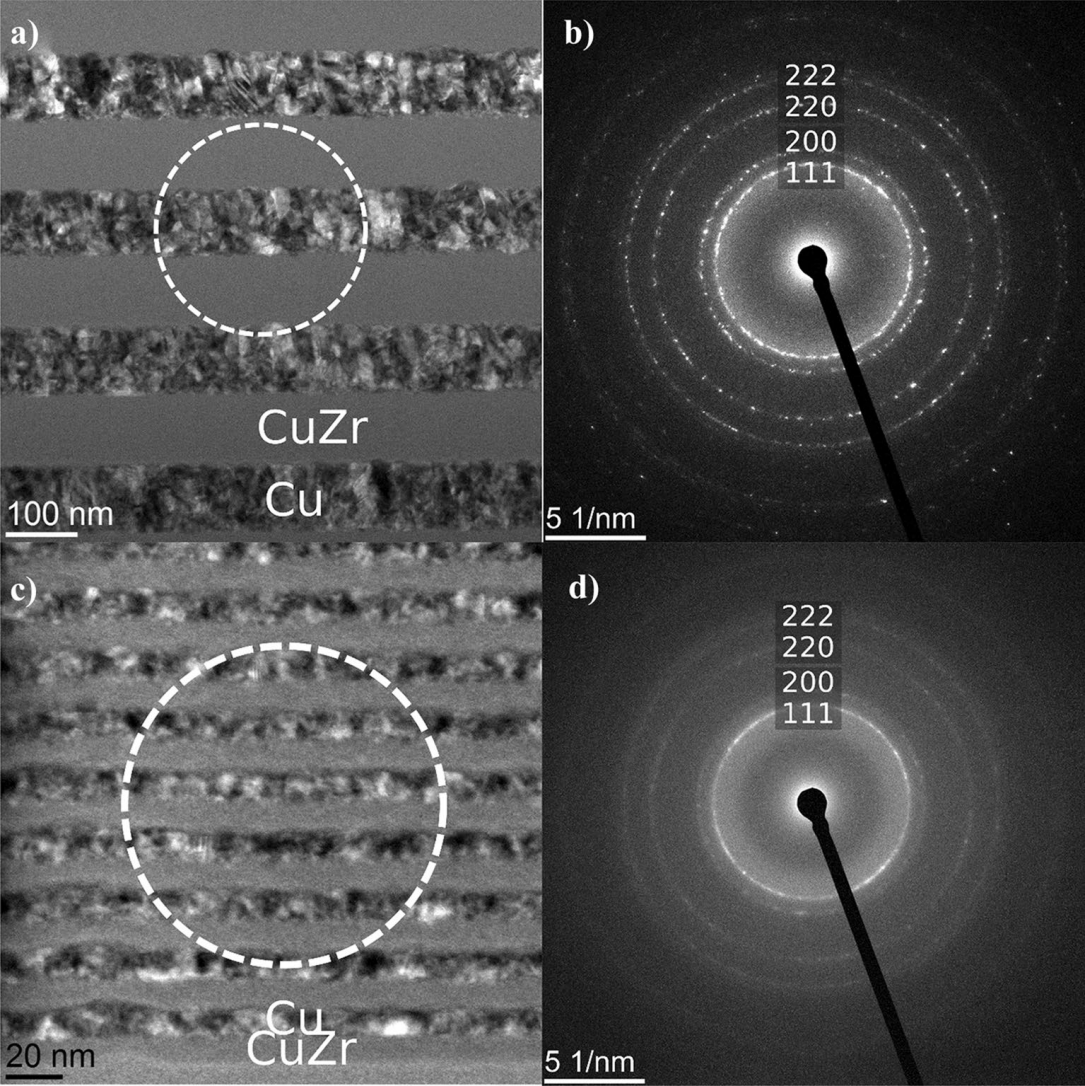
BF-STEM cross sectional micrographs of the investigated Cu-CuZr nanolaminates (a) BF-STEM micrograph of Λ_200_ multilayer and corresponding (b) selected area diffraction pattern (SAD). (c) BF-STEM micrograph of Λ_20_ multilayer and corresponding (d) SAD. The location of the selected area aperture is indicated by a dashed circle in Fig. 1(a) and (c), respectively.

Figure 1(a) shows the BF-STEM cross sectional micrograph of the Λ_200_ architecture, revealing homogeneous layer thickness throughout the growth direction and smooth interfaces separating the sublayers. The CuZr layers show a homogenous contrast indicative of their pure amorphous nature and the Cu layers show a nanocrystalline grain size. This is confirmed by the corresponding SAD pattern in Figure 1(b) showing sharp rings from the nanocrystalline Cu layers along with a broad ring stemming from the amorphous CuZr. The BF-STEM image of the Λ_20_ architecture [Figure 1(c)] shows similar microstructure but a smaller layer thickness. The corresponding SAD pattern in Figure 1(d) is again indicative of nanocrystalline Cu and amorphous CuZr. Both architectures have in common that they are dense (without pores) and that the deposition rates could be precisely adjusted.

### Nanoindentation

Young’s moduli and hardness values of all the samples were measured using nanoindentation with the Oliver and Pharr method (as described in the Methods section). Additionally, the hardness and Young’s modulus of the pure substrates were also measured with the same approach. For the glass substrate the hardness and Young’s modulus were found to be *H*_glass_ = 7.2 ± 0.4 GPa and *E*_glass_ = 79.0 ± 2.7 GPa, respectively, for the Si substrate the hardness and Young’s modulus were *H*_Si_ = 11.7 ± 0.2 GPa and *E*_Si_ = 174.1 ± 4.8 GPa, respectively.

**Figure 2:**
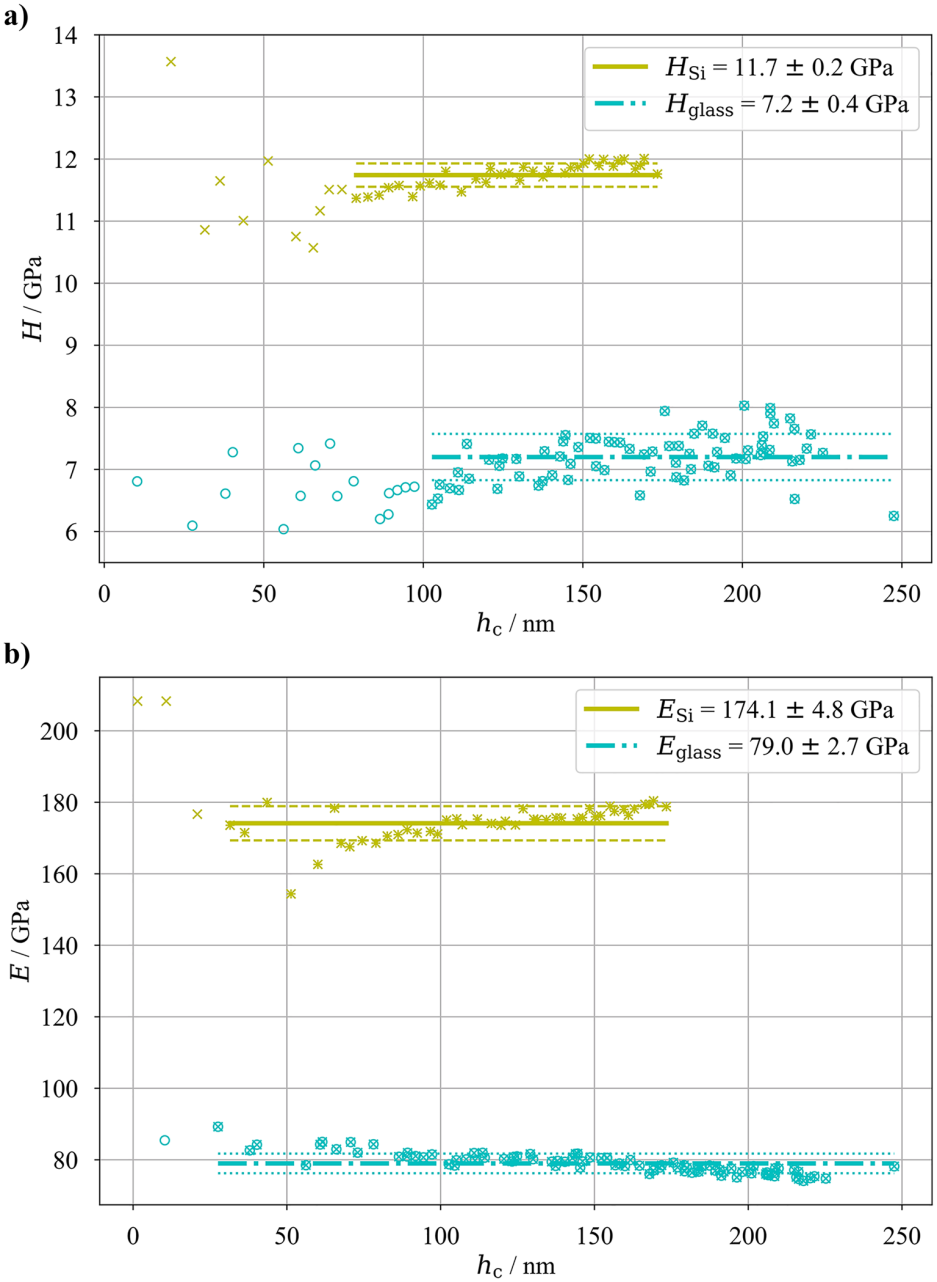
Hardness (a) and Young’s modulus (b) results from nanoindentation measurements of substrates as a function of indentation depth; simple “x” datapoints were excluded from the mean value evaluation.

**Figure 3:**
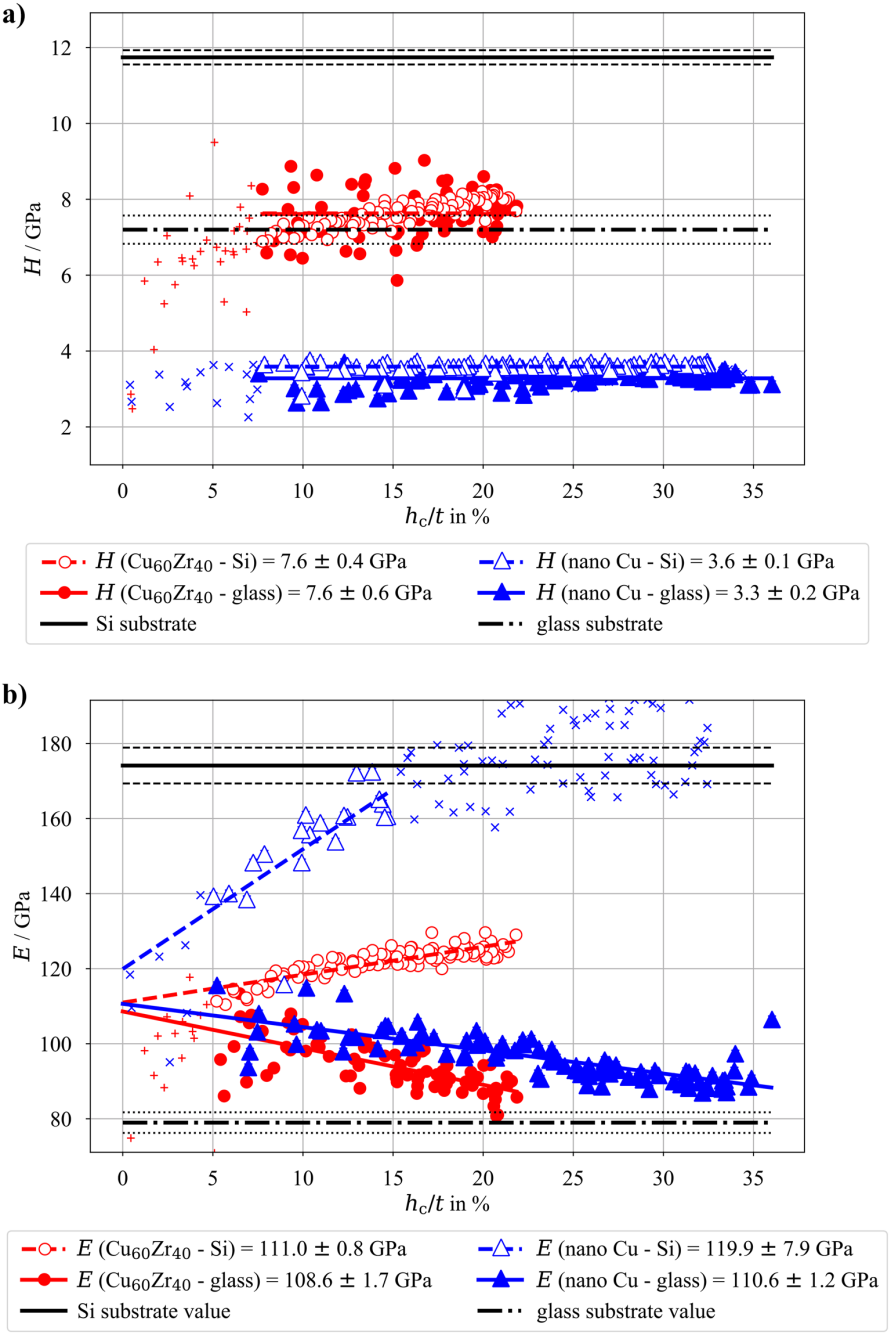
Hardness (a) and Young’s modulus (b) results from nanoindentation measurements as a function of contact depth relative to the film thickness (*h*_c_/*t*) of the monolithic films of amorphous Cu_60_Zr_40_ and nanocrystalline Cu on Si and glass substrates; the substrate values are depicted with their respective standard deviation boundaries.

**Figure 4:**
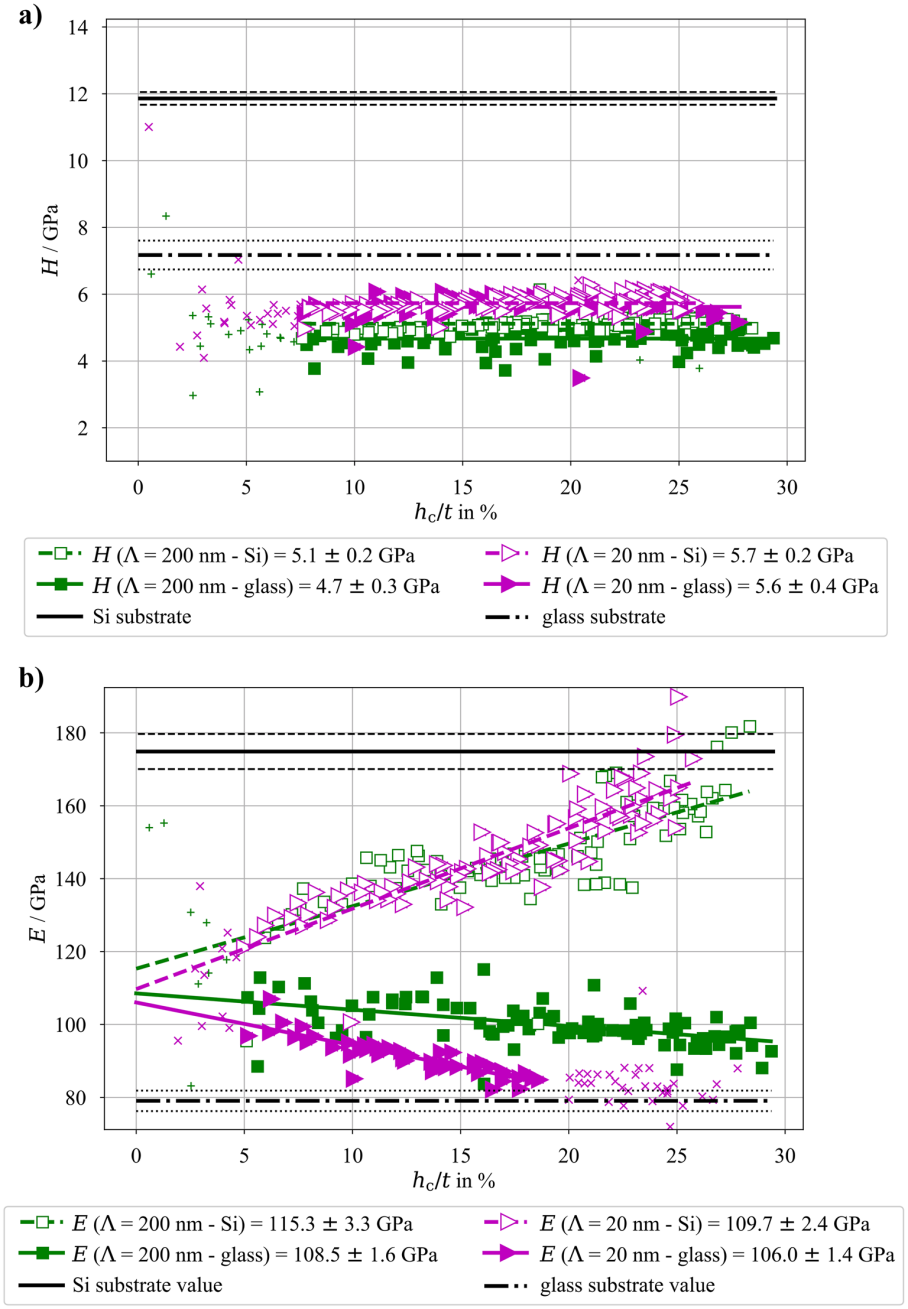
Hardness (a) and Young’s modulus (b) as a function of contact depth relative to the film thickness (*h*_c_/*t*) for both multilayer architectures (Λ = 20 nm vs. Λ = 200 nm) deposited onto Si and glass substrates, respectively; the Young’s modulus of the bare substrates are depicted with their respective standard deviation boundaries.

The resulting datapoints (hardness and modulus as a function of contact depth *h*_c_, as seen in Figure 2) showed very small scatter. For hardness measurements the low indentation depth indents were omitted to avoid the well-known indentation size effect (ISE, see e.g., [18–20]). For Young’s moduli evaluation, results exhibit constant behavior without any variations along with the indentation depth (as expected for bulk substrate materials) and only datapoints for contact depths below the calibration function validity limit were omitted from calculations. For simplicity, the substrate results shown in detail in Figure 2 will only be shown with their mean value and standard deviation as a reference in Figures 3 and 4, since the focus will then be on the results of the multilayers. Measured values of both substrates correspond well with available literature data. For Si the Young’s modulus is reported between 150 and 190 GPa (for < 111 > Si at room temperature) and hardness between 10 and 13 GPa (see e.g., [17, 21–24]). For sheet glass the Young’s modulus is reported between 70 and 100 GPa with hardness values ranging from 5 to 8 GPa (see e.g., [25–27]).

Since the monolithic layers and multilayers are expected to be influenced by a substrate effect (due to their relatively low film thickness), a more complex approach has to be used for the results evaluation, similarly to our previous work on Mo and MoTa thin films nanoindentations [4] expecting the apparent Young’s modulus to vary between the thin layer value (for *h*_c_ → 0 nm) and substrate value (for higher *h*_c_).

The absence of pile-ups—which are known to alter the nanoindentation results—for the four investigated thin film systems was confirmed using scanning electron microscopy and focused ion beam sectioning of the indented multilayer to study the indent morphology at higher magnification. We could confirm that no pile-up formation was created at the used indentation loads.

The Young’s moduli nanoindentation measurements of the monolithic amorphous CuZr and nano-crystalline Cu deposited onto glass and Si are depicted in Figure 3. Similar to the substrate datasets, the indents with low contact depth were omitted to obtain the hardness results due to possible indentation size effect and only the plateau values were used. The Young’s modulus measurements for small contact depths were omitted since the calibration function is not valid for such shallow indentation depth and the results are influenced by the sample surface roughness. Additionally, results for nano-Cu on Si substrate exceeding indentation depths *h*_c_ > 150 nm are showing pure substrate results due to the substrate effects, therefore, they were also excluded. The excluded results from all sets are marked by “ + ” and “ × ” symbols for the monolithic Cu and CuZr films, respectively, in Fig. 3, with color of the respective thin film material. The used datasets show clear transition between measured material value for *h*_c_ → 0 nm and substrate *E*-value for higher *h*_c_ for both materials. In the present case, the transition between low and high *h*_c_-values could be approximated with a linear function (in the form of *E* = *E*_0_ + *k*·*h*_c_, via Python scipy.optimize function). Assuming the real value of each thin film material is given by this linear fit for *h*_c_ = 0 nm—for such *h*_c_ there should be no substrate influence, the elastic moduli of each sample (the monolithic films on different substrates) could be evaluated from the *E*_0_ value obtained by the fit (with its standard deviation obtained from the fitting procedure) and they are shown in Figure 3. Additionally, since for *h*_c_ = 0 there is no substrate effect, the results of the samples with same monolithic thin film material but different substrate materials can be combined (assuming the Gaussian distribution of the evaluated *E*_0_, weighted averaging of the two mean values with their standard deviations could be performed), resulting in the Young’s modulus for Cu_60_Zr_40_ and nano-Cu as *E*_CuZr_ = 109.9 ± 1.8 GPa and *E*_nano-Cu_ = 112.2 ± 4.9 GPa, respectively. Comparing these values with literature data, the measured Young’s modulus for nanocrystalline Cu films have been reported to be between 100 and 130 GPa as determined via tensile tests in [28]. Young’s moduli of amorphous Cu50Zr50 thin films as a function of composition were determined in [15] leading to values of approximately 114 GPa and 107 GPa for thin film and ribbon samples, respectively. Both literature values are in line with our obtained nanoindentation results.

The hardness values for the monolithic Cu_60_Zr_40_ and nanocrystalline Cu is *H*_CuZr_ = 7.6 ± 0.5 GPa and *H*_nano-Cu_ = 3.4 ± 0.2 GPa, respectively, with only a negligible sign of substrate effects and comparable with available literature data [15, 28]. To confirm or reject the comparability of the samples on different substrates, a statistical *T*-test was performed to test the null H0-hypothesis (independent sample means are equal) with the α-value = 0.05. The resulting *p*-value for the Cu_60_Zr_40_ on Si and glass substrates was 0.7, which is larger than the α-value, meaning the H0-hypothesis could not be rejected (meaning there is no substrate effect). For the monolithic nano-Cu layer on Si and glass substrates, the *p*-value resulted in 9.2 × 10^–20^, therefore, the null hypothesis was rejected and there is some statistical difference between the hardness measurements for nano-Cu on Si and glass substrates, albeit very small. However, since the main objective of this manuscript is the Young’s modulus, the hardness of the monolithic films will not be discussed in more detail.

As a next step, a proper characterization of both multilayer architectures (with bilayer thickness Λ = 20 nm vs. Λ = 200 nm) can be performed. Since the only difference between these two architectures is the sublayer thickness (10 nm vs. 100 nm), but having the same stack thickness and ratio between CuZr and Cu, it can be assumed that both architectures should exhibit the same apparent Young’s modulus, depending only on the used substrate. This is clearly visible in Figure 4, where all four combinations (both multilayer architectures deposited onto glass and Si substrates, respectively) are plotted together.

Results presented in Figure 4 were processed in a similar way as ones from Figure 3—low contact depths in hardness measurements were excluded due to the indentation size effect, low contact depth measurements for Young’s modulus evaluation were not used due to the uncertainty in the calibration area function. Additionally, the indentation into Λ = 20 nm multilayer architecture with *h*_c_/*t* higher than 20% were not used since they only represent the elastic material properties of the substrate.

Nanoindentation of both multilayer systems on the Si substrate exhibit indistinguishable Young’s modulus values as a function of indentation depth, while the samples on glass substrate show a slightly different strength of substrate effect, but reach a similar value when *h*_c_ → 0 nm. The reason that the substrate effect is more pronounced and starts more dramatically for lower *h*_c_-values for the films deposited on glass substrate, can be explained by the fact that glass is more compliant than Si.

The convergence of the results to one *E*-value when *h*_c_ → 0 nm regardless of the multilayer structure and used substrate (visible in Figure 4) shows that the Young’s modulus of the multilayer as a whole is more influenced by the relative composition rather than the number of interfaces and individual layer thicknesses. With this in mind, the average of the individual values from the four systems (as depicted in Figure 4) can be used to deduce a combined Young’s modulus of the Cu_60_Zr_40_/nano-Cu multilayer as *E*_Cu60Zr40/nano-Cu_ = 110.4 ± 4.0 GPa. For the estimation of the final Young’s moduli, the approach with linear fitting and evaluation of* E*_0_-values for each subset was used. Then the weighted averaging of the four mean values with their standard deviations could be performed, assuming again their individual Gaussian distributions, similarly as for monolithic layers.

The hardness of the measured multilayers resulted in values differing between the two investigated architectures but the substrate material had less impact on the hardness values for the same multilayer specimens. Therefore, in contrast to the *E*-value, the hardness is influenced by the bilayer thickness of the multilayer architecture resulting in: *H*_Λ=200 nm, glass_ = 4.7 ± 0.3 GPa, *H*_Λ=200 nm, Si_ = 5.1 ± 0.2 GPa, *H*_Λ=20 nm, glass_ = 5.6 ± 0.4 GPa, and *H*_Λ=20 nm, Si_ = 5.7 ± 0.2 GPa. Since the intervals of the mean values of all four measured material systems seem to be very close to overlap, a statistical *T*-test was performed to test the null H0-hypothesis (independent sample means are equal) with the α-value = 0.05. At first, similarly to the monolithic layers, the substrate influence on hardness measurements was tested. For the Λ = 20 nm system, the *p*-value resulted in 0.12, therefore the H0-hypothesis cannot be rejected and no substrate influence is observed. However, for the Λ = 200 nm system, the *p*-value resulted in 3.5 × 10^–18^, meaning that there is statistical difference between hardness measurements of this system on different substrates, albeit very small. More importantly, the difference between the Λ = 20 nm and Λ = 200 nm systems on same substrates was tested. The comparisons for Si and glass substrates lead to *p*-values of 8.3 × 10^–33^ and 1.1 × 10^–34^, respectively, which is way below the considered α-value, meaning that the H0-hypothesis could be rejected and there is a significant statistical difference between the hardness measurements of the Λ = 20 nm and Λ = 200 nm systems. As expected, increased hardness values for multilayer architectures with decreased sublayer thickness (i.e., increase of interface density) were measured, which is in agreement with previous works [29] and will not be further discussed here.

## Discussion

While nanoindentation has been widely employed to determine the mechanical properties of relatively thick metallic films attached to rigid substrates, an accurate determination of the Young’s modulus values of thinner films is still highly influenced by the underlying substrates. While the hardness measurements are in general reliable even for rather deep indentation depths (safely up to indentation depth of 1/5 of the film thickness for standard ceramic substrate—metallic thin film combination [4]), the elastic modulus of thin films can be measured precisely only with shallow, nanometer-regime indents, which are strongly influenced by various other factors. Therefore, we suggest an experimental strategy where the same coatings were deposited onto two distinct rigid substrates, with different mechanical properties to decouple the substrate effect on the nanoindentation, namely glass (*E*_glass_ = 79.0 ± 2.7 GPa, *H*_glass_ = 7.2 ± 0.4 GPa) and silicon (*E*_Si_ = 174.1 ± 4.8 GPa, *H*_Si_ = 11.7 ± 0.2 GPa).

The four examined systems were subjected to a large number of nanoindentation experiments with different loads. As expected the deep indents are affected by the underlying substrates (see Figures 3 and 4) and the evaluated Young’s moduli for deep indents clearly converge toward the respective substrate value. However, shallower indents start to deviate from the substrate value and show a linear increase of the coatings influence with decreasing contact depth. This effect extends toward *h*_c_ = 0 nm, while samples where the substrate elastic modulus is lower than that of the coating exhibit an increase in measured apparent Young’s modulus and samples with stiffer substrate than that of the coating exhibit a decrease in measured values.

One can clearly see, that regardless of the substrate, the apparent Young’s modulus obtained via nanoindentation converges to one value for each coating system when extrapolated to *h*_c_ = 0 nm. Namely, the indentation of the nano-Cu deposit leads to *E*_nano-Cu_ = 112.2 GPa and for the Cu_60_Zr_40_ deposit leads to *E*_CuZr_ = 109.9 GPa. With a similar approach both Cu -CuZr multilayered architectures (with Λ = 20 nm and Λ = 200 nm were examined, leading to similar Young’s moduli values for both architectures (see Figure 4). Since the individual layers of the two multilayer systems show identical microstructure and both systems exhibit the same overall volume fraction of Cu and CuZr, only the number of interfaces is changed. Therefore, it can be concluded that the number of interfaces (or interface density) has no impact on the measured Young’s modulus of the whole multilayer. Therefore, the values for elastic modulus obtained for the system with Λ = 20 nm and Λ = 200 nm can be combined into one, general value of *E*_Cu60Zr40/nano-Cu_ = 110.4 GPa (with the use of average mean value and standard deviation, assuming the Gaussian distribution of the results). A simplified and widely used “rule of mixture” also assumes that the Young’s modulus of a composite is only dependent on the relative portion of individual materials in the multilayer. Knowing the Young’s modulus of each material involved in the examined multilayers (see Figure 3) and the multilayers’ composition, the “rule of mixture” leads to *E*_mix_ = 109.5 GPa. This value is slightly lower than the measured combined value of *E*_Cu60Zr40/nano-Cu_ = 110.4 GPa (combination of nanoindentation of both Λ = 20 nm and Λ = 200 nm multilayer architectures), however, still within the statistical range of measured results.

The resulting elastic moduli of both monolithic materials and multilayered thin films can be also compared with available literature. The values with their respective standard deviations are presented in Table 1 and show a good agreement between measured results and older works on similar materials.

**TABLE 1:** Comparison of Measured Young’s Modulus Results of the Same Material Systems with Literature.

	*E*_Cu60Zr40_/GPa	*E*_nano-Cu_/GPa	*E*_Cu60Zr40/nano-Cu_/GPa	*E*_mix_/GPa
Current work	109.9 ± 1.8	112.2 ± 4.9	110.4 ± 4.0	109.5 ± 2.9
Guo et al. [14]	40–90	–	–	–
Rauf et al. [15] (thin film)	114.3 ± 2.244	–	–	–
Rauf et al. [15] (ribbon)	106.7 ± 6.984	–	–	–
Yu and Spaepen [28]	–	100–130	–	–
Guo et al. [12] (10 nm Cu)	–	–	100 ± 2	–
Guo et al. [12] (100 nm Cu)	–	–	98 ± 2	–

The presented data clearly show that the substrate influence on the elastic material properties measurements via nanoindentation is largely pronounced, as previously reported in [4]. Comparison of measured values of apparent Young’s modulus for *h*_c_/*t* = 10% (historically assumed “safe indentation depth”) leads to elastic modulus of Cu_60_Zr_40_ around 100 GPa for sample with glass substrate and around 120 GPa for sample with Si substrate, elastic modulus of nano-Cu around 107 GPa for sample with glass substrate and around 150 GPa for sample with Si substrate. Similarly, the multilayered architectures yield at *h*_c_/*t* = 10% the value of 100 GPa and 130 GPa for glass and Si substrates, respectively. This leads to errors ranging between 10 and 30%, which should be unacceptable. The presented method therefore shows a clear, straightforward method to evaluate a thin film’s Young’s modulus with self-controlling mechanism in terms of convergence of the nanoindentation results toward one value, regardless of the used substrate.

The extend of the substrate effect and its difference throughout the results can be explained (for the case of the monolithic films) by the difference between yield limits of the used materials, when the materials yielding points are largely different. The Oliver-Pharr method uses the unloading portion of the load–displacement indentation curve, therefore measuring only the elastic response of the indented material. It is thus obvious, that when combined, materials with lower yield limit will exhibit relatively less pronounced elastic response than materials with higher yield limit (at the same load level), because the more ductile material will have relatively larger plastic zone, therefore will not participate in measured “spring-back” of the material. Therefore, Cu on Si substrate are expected to have a larger substrate influence than Cu on glass substrate. It should be noted that according to Tabor [30], the yield limit of a material is usually proportional to its hardness. Since the hardness measurements accompanied the nanoindentation presented in this work, the hardness values will be used onward.

When comparing the ratios of film to substrate hardness, the largest value of 1.06 is yielded by the combination of Cu_60_Zr_40_ and glass, the lowest value is 0.29 for nano-Cu on Si, while Cu_60_Zr_40_ on Si leads to 0.65 and nano-Cu on glass to 0.48. Indeed, the monolithic nano-Cu film on Si substrate shows the most pronounced substrate effect with pure substrate measurements around *h*_c_/*t* = 10% (see Figure 3) while having largest relative difference between the different materials’ hardness values. The nano-Cu on glass and Cu_60_Zr_40_ on Si follow with smaller substrate effects (the pure substrate measurements should appear approximately at *h*_c_/*t* equal to 40% and 55%, respectively), in-line with the hardness ratios. However, for the case of Cu_60_Zr_40_ and glass combination, the hardness values of the materials are almost equal, therefore, the yield phenomenon is not pronounced. Instead, the elastic mismatch between the two materials starts to be the main reason for the substrate effect. With increasing load, the thin coating starts to bend under the indenter tip and it “sinks” into the more compliant substrate. However, it has to be noted that such a simplified explanation by either hardness or elastic mismatch is not complex enough for a general description of the substrate effect extent. In reality, their combination affects the overall measured apparent elastic modulus and for multilayer systems other parameters may play a significant role (e.g. the interface density and related phenomena). However, for the case of the presented monolithic films, this simplified comparison works and shows that even small variances in mismatch between film and substrate materials lead to high substrate effect during elastic modulus measurements. For a more generalized description, a more thorough research will be conducted.

It has to be noted that the presented results were obtained on the material systems with only one overall coating thickness and with a small range of film-to-substrate hardness and elastic moduli ratios (0.3 to 1.1 for hardness and 0.6 to 1.5 for elastic moduli). Therefore, without further research, this method is for the time being valid only in this material properties range. However, more material systems with a larger variety of the material properties are under the research and are subject to future publications.

## Conclusion

While the measurement of elastic material properties via nanoindentation of thin film structures attached to rigid substrates is still challenging, the presented results show that it is possible to reliably evaluate the Young’s modulus of 1 µm thick amorphous and nanocrystalline coatings as well as amorphous-crystalline multilayers. This is achieved by extrapolation from a large set of indents with different contact depths. To ensure that the substrate effect is fully excluded and thus obtain an accurate Young’s modulus, the same material is deposited on rigid substrates with different mechanical properties, one with significantly lower and the other with higher *E*-values than what is initially predicted for measured thin film material. A good agreement is reached for the two different substrates, demonstrating the reliability of the technique, assuming the film-to-substrate hardness and elastic moduli ratios in range of 0.3 to 1.1 and 0.6 to 1.5, respectively. For the monolithic layers the values can be compared to literature data showing good agreement. While it is well-known that the hardness of amorphous-crystalline multilayers strongly increases with the number of layer thickness, measurements of the Young’s modulus are sparse. Therefore, we compared a model Cu–CuZr multilayer system with equal total thickness but different bilayer thickness. Our experiments demonstrate that the interface density has an influence on the hardness but not on the Young’s modulus of the composite.

## Methods—experimental procedures

### Investigated materials systems/Thin film synthesis

**Figure 5:**
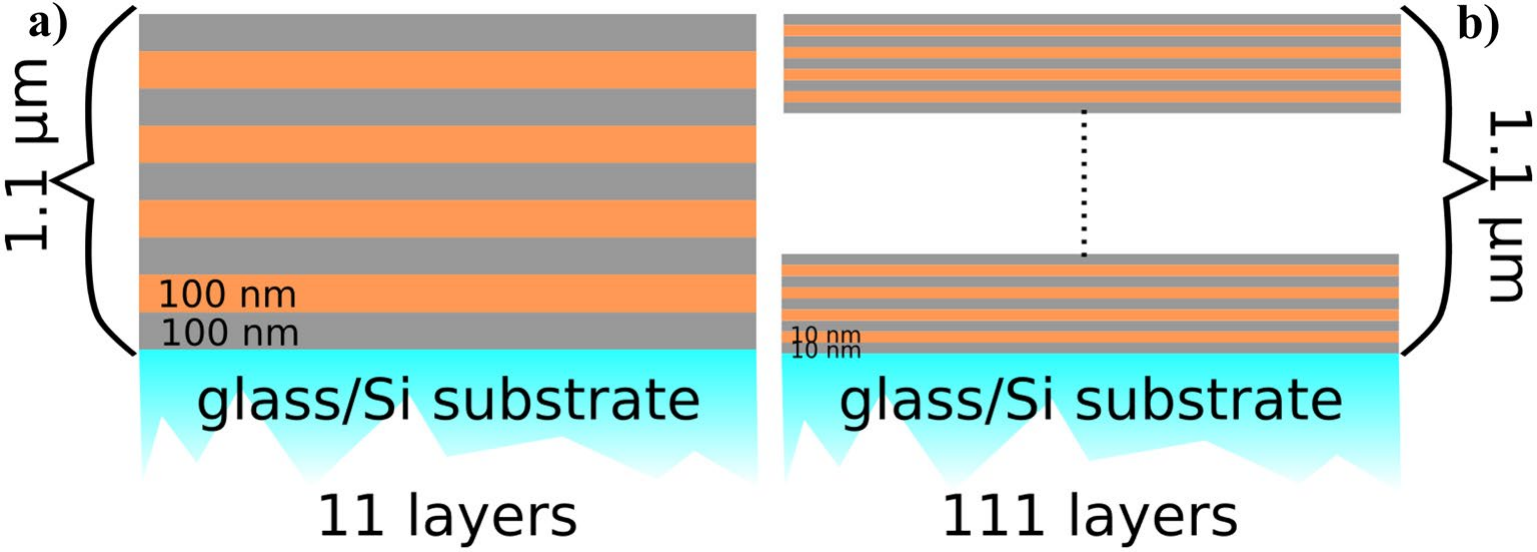
Schematic of studied multilayer-architectures (a) Λ_200_-multilayer with a periodic bilayer thickness Λ = 200 nm (total of 11 layers) and (b) Λ_20_- multilayer with bilayer thickness Λ = 20 nm layer thickness (total of 111 layers).

Two different amorphous crystalline nanolaminate architectures composed of alternating amorphous Cu_60_Zr_40_ (called CuZr hereafter) and nanocrystalline Cu of same sublayer thickness are compared. The nanolaminates were designed to have the same global multilayer stack thickness of approximately 1 µm but differ in their periodic bilayer thickness (Λ = 20 nm vs. Λ = 200 nm) referred to as Λ_20_- multilayer and Λ_200_-multilayer, respectively. The Λ_200_-multilayer contains 11 layers and the Λ_20_-multilayer 111 layers, as schematically shown in Fig. 5. To obtain comparable substrate-thin film interface qualities and roughness for both architectures, the CuZr layer was the initial layer closest to the substrates and the top layer of both stacks. For comparison, 1 µm thick monolithic films of A-CuZr and NC-Cu were deposited using the same sputtering parameters used for the previously described multilayers. In total four different film types (2 multilayers and 2 monolithic thin films of nanocrystalline Cu and amorphous CuZr) with the same global film thickness of approximately 1 µm were studied. To decouple the substrate effect on the nanoindentation results, all four film types were deposited onto glass and Si substrates within the same deposition runs. These substrates were chosen as for glass the Young’s modulus and hardness values are lower and for Si these values are expected to be higher than the values expected for the investigated thin films.

All thin film samples of this study were synthesized using direct current magnetron sputtering within a lab-scale modular PVD deposition chamber equipped with two sputtering sources (Korvus Technology) using two elemental targets with a diameter of 50.8 mm and a thickness of 3.2 mm (a 99.999% Cu target and a 99.95% Zr target, both provided by HMW Hauner). Prior to sputtering the base pressure of the deposition chamber was set to below 2.6 × 10^–5^ mbar and throughout all depositions the working gas (Ar) pressure was set to 1.7 × 10^–3^ mbar. The substrates were attached to a sample stage rotating with a frequency of 20 rounds/minute during the deposition runs. The Cu films were deposited at 40 W target power yielding sputtering rates of 10 nm/min. The amorphous CuZr films were synthesized using co-deposition of both pure metal targets, where the Cu target power was set at 40 W and the Zr target power was set to 95 W, leading to a deposition rate of 20 nm/min. Thus, the different thicknesses of the Cu and CuZr layers were achieved by adjusting the deposition durations.

### Microstructural characterization

The cross-sectional microstructures of both multilayer architectures (Λ = 200 nm vs. 20 nm) were studied in a JEOL 2200FS transmission electron microscope (TEM) operating at 200 kV. Electron transparent lamellae of each architecture were prepared using a focused ion beam (FIB) liftout technique within a dual beam Zeiss Auriga FIB workstation equipped with an Omniprobe micromanipulator. BF-STEM (bright field scanning transmission electron microscopy) micrographs were obtained to reveal the microstructure of the multilayers. In addition, selected area diffraction (SAD) patterns were recorded for both architectures using a selected area aperture with a diameter of 400 nm and 200 nm, for the Λ_200_ and Λ_20_-architecture, respectively.

### Nanoindentation

The mechanical properties of the Λ_200_ and Λ_20_ multilayer architectures and their monolithic counterparts (nanocrystalline Cu and amorphous Cu_60_Zr_40_, respectively) deposited on glass and Si substrates were probed using a TS77 Select Bruker-Hysitron nanoindentation platform and a well-calibrated Berkovich tip. The area function and frame compliance calibrations were made using 100 Open-Loop (OL) indents into fused silica for each calibration run with a 10 s load—5 s hold—10 s unloading profile and maximum loads between 100 µN and 10 mN, resulting in the calibrated area function for indentation depths h_c_ between 7 and 200 nm. Quasi-static indents into both multilayer architectures, and the monolithic Cu and Cu_60_Zr_40_ films deposited in a similar fashion onto Si and glass substrates were performed. To avoid strain- or load-rate effects [31], a constant load rate of 200 µN/s was used for all indents.

A total of 100 force-controlled indents in a regular pattern with 10 µm spacing were made into each thin film type on both substrates, respectively, with indentation loads varying between 25 µN and 10mN. This approach provided sets with a statistically significant number of datapoints while also allowing to evaluate the dependency of results on the contact depths during nanoindentation. Furthermore, nanoindentation measurements were also conducted on the bare glass and Si substrates without films to confirm their mechanical properties (as a reference point), the same procedure as for the thin films nanoindentation was used.

All of the resulting load–displacement curves were analyzed using the standard Oliver and Pharr method [32] and the calibrated area function. The elastic modulus of the indented material, *E*, was evaluated from the reduced elastic modulus, *E*_r_, obtained via nanoindentation using Eq. (1):

1 $$\frac{1}{{E}_{\mathrm{r}}}=\frac{1-{\nu }_{t}^{2}}{{E}_{\mathrm{t}}}+\frac{1-{\nu }^{2}}{E}$$

where *E*_t_ and *ν*_t_ is Young’s modulus (1140 GPa) and Poisson’s ratio (0.07), respectively, of the indenter material [33–35] and *E* and *ν* are Young’s modulus and Poisson’s ratio of the indented materials, respectively.

## Data Availability

The raw data will be provided by authors or the research institution upon a reasonable request.

## Code availability

The code used for evaluation will be provided by authors upon a reasonable request.
